# Reply to: Statins in esophageal variceal bleeding: The right data for the right research question

**DOI:** 10.1097/HC9.0000000000000733

**Published:** 2025-06-19

**Authors:** Nina Kimer, Rasmus H. Gantzel, Thit M. Kronborg

**Affiliations:** 1 Gastro Unit, Medical Division, Hvidovre University Hospital, Hvidovre, Denmark; 2 Department of Clinical Medicine, Faculty of Health Sciences, University of Copenhagen, Copenhagen, Denmark; 3 Department of Hepatology and Gastroenterology, Aarhus University Hospital, Aarhus, Denmark; 4 Department of Gastroenterology, Copenhagen University Hospital—North Zealand, Hillerød, Denmark

We read with great interest the letter by Zyngier et al^1^ presenting preliminary data from a randomized controlled trial investigating the efficacy of adding rosuvastatin in patients with clinically significant portal hypertension and insignificant response to non-selective beta-blockers (NSBBs).

The authors hypothesize that the potential effects of statins on portal hypertension depend on prior variceal bleeding and the severity of decompensated cirrhosis.

The potential efficacy of statins to reduce the risk of hepatic decompensation and complications associated with portal hypertension has been explored in recent years.^2^,^3^ Clinical trials are preferably designed to mirror the clinical reality, and hence, a great variation in included patients must be accounted for in the analysis. In the StatLiver trial conducted between 2019 and 2022, only 18 of 78 included participants received NSBBs at baseline. In the statin group, 3 were for primary prophylaxis and 2 for secondary prophylaxis of variceal bleeding. In the placebo group, 8 were for primary prophylaxis and 5 for secondary prophylaxis. Only 4 participants experienced variceal bleeding during trial follow-up. In the placebo group, one had a variceal bleeding without prior NSBB treatment, and one had a variceal bleeding despite prior NSBB treatment. In the statin group, one had a variceal bleeding and one bled from a Dieulafoy lesion; none of them had prior NSBB treatment.

The StatLiver trial was unfortunately ended before the sample size was reached, and small numbers in subgroups complicate stratified analyses. The heterogeneity of included participants in clinical trials may limit our understanding of the efficacy and timing of statins. Further elaboration on when to initiate statins, the peak efficacy of statins, and the interaction with NSBBs will be elucidated in the coming years as more clinical trials are on their way.

We encourage authors to share patient-individual data for meta-analyses, as this will aid our understanding of the anti-hypertensive effects of statins, just as registry studies from larger population-based cohorts may be valuable in assessing subgroup outcomes in portal hypertension and cirrhosis. High-quality research and the future will supply clinical answers.
